# Colonic Stents for Colorectal Cancer Are Seldom Used and Mainly for Palliation of Obstruction: A Population-Based Study

**DOI:** 10.1155/2016/1945172

**Published:** 2016-03-29

**Authors:** Anna M. Borowiec, Charlie S. K. Wang, Elaine Yong, Calvin Law, Natalie Coburn, Rinku Sutradhar, Nancy Baxter, Lawrence Paszat, Jill Tinmouth

**Affiliations:** ^1^Department of Surgery, University of Toronto, Toronto, ON, Canada; ^2^Department of Medicine, University of Toronto, Toronto, ON, Canada; ^3^Dalla Lana School of Public Health, University of Toronto, Toronto, ON, Canada; ^4^Department of Radiation Oncology, University of Toronto, Toronto, ON, Canada

## Abstract

Self-expandable stents for obstructing colorectal cancer (CRC) offer an alternative to operative management. The objective of the study was to determine stent utilization for CRC obstruction in the province of Ontario between April 1, 2000, and March 30, 2009. Colonic stent utilization characteristics, poststent insertion health outcomes, and health care encounters were recorded. 225 patients were identified over the study period. Median age was 69 years, 2/3 were male, and 2/3 had metastatic disease. Stent use for CRC increased over the study period and gastroenterologists inserted most stents. The median survival after stent insertion was 199 (IQR, 69–834) days. 37% of patients required an additional procedure. Patients with metastatic disease were less likely to go on to surgery (HR 0.14, 95% CI 0.06–0.32, *p* < 0.0001). There were 2.4/person-year emergency department visits (95% CI 2.2–2.7) and 2.3 hospital admissions/person-year (95% CI 2.1–2.5) following stent insertion. Most admissions were cancer or procedure related or for palliation. Factors associated with hospital admissions were presence of metastatic disease, lack of chemotherapy treatment, and stoma surgery. Overall the use of stents for CRC obstruction remains low. Stents are predominantly used for palliation with low rates of postinsertion health care encounters.

## 1. Introduction

Colorectal cancer (CRC) is the third most common malignancy in North America and it is the second most frequent cause of cancer-related mortality in both men and women [1, 2]. In up to 20% of patients, the tumor results in colonic obstruction [3–6]. Historically, surgery (resection or diverting stoma) was the only treatment option for CRC obstruction. However, an emergency operation in this setting carries 18–46% risk of morbidity, 16–38% mortality [3, 4, 7–9], high likelihood of stoma creation especially in left-sided cancers [8], and low rates of subsequent stoma closure [10]. In the early 1990's, self-expanding metallic stents were introduced as an alternative to surgery in palliative patients with CRC obstruction [11, 12] and were subsequently extended to patients with curable obstructing CRC as a bridge to definitive surgery [13].

The use of colorectal (CR) stents in the palliative setting avoids a major operation and the need for stoma creation while shortening hospital stay and the time to administration of palliative chemotherapy [14]. By extension, when used as a bridge to surgery in patients with curable CRC, CR stents allow for bowel decompression, making a subsequent one-stage curative operation more technically feasible, allowing for the optimization of hydration and nutritional status and for preoperative cancer staging [15–18].

In retrospective single center studies summarized by Sebastian et al., stent insertion for palliation has over 90% technical (adequate procedural stent deployment) and clinical (relief of obstructive symptoms) success rates; in the bridge to surgery patients, similar technical but lower clinical (71%) success rates are reported [19, 20]. In the same studies, the overall complication rate was 25% in both types of patients [19, 20]. Results from randomized controlled trials of stents for acute obstruction due to CRC have been less impressive than the initial retrospective studies. In a meta-analysis of controlled trials comparing colonic stentingversus surgical decompression for obstructing colorectal cancers, surgery was found to be more clinically successful than stenting and rates of complications and mortality were, at best, similar [21]. Two multicentered randomized controlled trials of stents versus surgery for acute obstruction were prematurely terminated due to unacceptably high complications associated with stent deployment [22, 23]. These data raise important questions for the role of CR stents in the management of acute obstruction; however, their exact role in palliative patients with CRC remains less clear due to limited data from controlled studies in this population [24, 25]. To date, studies have been performed in highly specialized tertiary care centers, there is, a paucity of data on stent use in “real-world settings.”

In the current study, we performed a population-based analysis of stent use for CRC obstruction in the province of Ontario. The aims of the study were to (1) describe the characteristics (patient, provider, and institution) of colonic stent utilization in patients with CRC and (2) to report health outcomes and health care encounters after stent insertion in the usual clinical practice across the province of Ontario.

## 2. Methods

We conducted a retrospective population-based cohort study of persons with CRC who received a CR stent in the province of Ontario from April 1, 2000, to March 30, 2009.

### 2.1. Data Sources

The study was conducted at the Institute for Clinical Evaluative Science (ICES), which contains health records for all residents of Ontario. These records are held in administrative databases that are linked by an encrypted version of each resident's provincial health plan number. For the purpose of this study six databases were used: (1) the Ontario Cancer Registry (OCR), which has captured cancer diagnoses and deaths since 1964 and is estimated to be 95% complete [26]; (2) the Canadian Institute for Health Information (CIHI) Discharge Abstract Database (CIHI-DAD), which contains data on hospital admissions in Canada; (3) the CIHI National Ambulatory Care Reporting System (CIHI-NACRS), which includes information on patient visits for hospital based ambulatory care, including emergency department visits and day surgeries; (4) the Registered Persons Database of Ontario (RPDB), which contains demographic information for all eligible residents in Ontario; (5) the Ontario Health Insurance Plan (OHIP) Physicians Claims Database, which comprises all physician billing records, including endoscopy and radiology; and (6) the ICES Physician Database (IPDB), which contains physician information.

### 2.2. Study Cohort

Using the OCR database, all cases of CRC from 1964 onwards were identified, excluding cancer of the appendix and anus. Using OHIP procedure fee codes, both endoscopic (code E630 or E641, defined as endoscopic placement of stent in colon or rectum, resp.) and radiologic (code J059, defined as radiologic nonvascular stenting) stent insertions were identified between April 1, 2000, and March 31, 2009. Patients with an OHIP billing claim for stent insertion during this period of time and a concurrent or prior diagnosis for CRC were assumed to have colonic stent placed for treatment of CRC. To account for insertion of colonic stents in persons with newly diagnosed CRC, OCR registrations for up to six months after the OHIP stent insertion date were included to allow sufficient time for new CRC cases to be entered into the OCR.

All patients with a diagnosis of any noncolorectal cancer in OCR from five years prior to six months after stent insertion were excluded to minimize the possibility that a stent was placed for tumors other than CRC. As the code for radiologically inserted stents is applicable to all nonvascular stents, an algorithm was developed to exclude noncolorectal sites of stent insertions. Patients were included if they had CIHI-DAD CCP and CCI procedural codes related to a therapeutic intervention of the large intestine or rectum without a concurrent procedural code for a therapeutic intervention outside the large intestine or rectum within ±30 days of the OHIP J059 billing service date. Patients with missing DAD CCP and CCI procedural codes were also excluded.

Patients residing in the Southeastern Ontario health region were excluded as some physicians in this region are paid through alternate funding plans, which may affect completeness of OHIP billing claims. Over the study period, of the 51,251 patients diagnosed with colorectal cancer in the province of Ontario 4.8% were from the Southeastern health region.

### 2.3. Patient Characteristics and Other Covariates

Patient sex, median neighborhood income quintile, age at CRC diagnosis, and age and comorbidity at the time of index stent insertion were collected. Comorbidity was derived from CIHI hospital discharge diagnosis data for the 5 years preceding the index stent date using the Deyo adaptation of the Charlson Comorbidity Index score [27–29]. As all patients in the cohort had cancer and some had metastatic disease, this score was modified to exclude the two cancer-related categories (primary cancer and metastatic disease) and was classified as 0, 1, 2, or ≥3. A separate variable was created for the presence of metastases (if the Deyo/Charlson Comorbidity Index category for metastatic disease was positive or if there was a CIHI-DAD discharge diagnosis indicating metastatic disease within 6 months after stent insertion date). Other variables included specialty of the physician inserting the stent and type of hospital (community versus academic) where the stent was inserted. An academic hospital was defined as a hospital affiliated with a teaching institution. Fourteen out of 176 acute care hospitals in Ontario met this definition.

### 2.4. Outcome Definitions

Outcomes of interest included health outcomes (death, dilatation and repeat stenting, surgery, and chemotherapy) and health care encounters (emergency department visits and hospitalization). Death status and date of death were determined from the RPDB and OCR. Patients without a death registration in RPDB or OCR were considered alive at the end of follow-up (March 31, 2009). In order to account for migration out of province and out of hospital deaths not captured by RPDB or OCR, patients were excluded from all poststent survival analysis if they had no contact with the health care system, based on RPDB data, in the three years prior to the end of follow-up (March 31, 2009). A three-year time frame was chosen as three-to-five-year period is the recommended interval for follow-up colonoscopy after CRC resection. Patients alive and residing in Ontario would have been expected to come into contact with the health care system within that period of time. Using OHIP, all potential colonic dilatations (using Z513 and J065 for endoscopic and radiologic dilatations, resp.) and colonic stenting procedures after the index stent insertion date were identified. The algorithm described earlier was applied to radiologic codes in order to exclude noncolorectal dilatations and/or stenting. Patients who underwent abdominal surgery for CRC were identified using procedural codes in CIHI-DAD. Surgeries were classified as either those involving resection of a segment of colon or rectum and/or those involving creation of a stoma using a scheme adapted from the 2008 ICES Atlas of Cancer Surgery in Ontario [30]. Patients who received chemotherapy were identified using OHIP (codes G281, G381, G339, G345, and G359). Emergency department visits were identified using OHIP Physician Claims Database (prior to April 1, 2001) and CIHI-NACRS (after April 1, 2001). Data on length of stay and most responsible diagnosis were collected for all hospitalization following index stent insertion using CIHI-DAD. In addition, admissions for obstruction and/or perforation were identified with data from CIHI-DAD using modified versions of a previously published definition [31].

## 3. Data Analysis

The number of unique patients who received stents overall and by year was calculated. Patient and provider characteristics by mode of stent insertion and as well type of surgery after stent insertion by presence and absence of metastases were determined. The number and outcomes of patients admitted with obstruction and/or perforation after stent insertion were evaluated. Continuous data were compared using Student's *t*-test or Wilcox Rank Sum test, while categorical data were compared using Chi-square test or Fisher's Exact test, depending on the distribution of the data.

Time to death was examined using the Kaplan Meier approach. The survival curves were stratified by mode of stent insertion. Cumulative incidence curves that account for death being a competing risk were used to model time to nondeath events: surgery, repeat stent placement of dilation, and stoma creation [32]. To investigate the relationship between these time to nondeath events and various covariates (patient age, sex, metastases at presentation, and types of stent), we used a regression model under a cause-specific hazard approach to accommodate for the competing risk of death [32]. Subjects in the cohort were followed from index stent insertion until the outcome of interest and were censored if they did not have the outcome as of March 31, 2009.

The pooled rate of emergency department visits and hospital admissions was calculated by dividing the total number of emergency department visits and hospitalization that occurred in the total cohort by the total number of person-years of follow-up. Similarly, the pooled number of days spent in hospital per person-year was calculated by dividing the total number of inpatient hospital days by the total number of person-years of follow-up. A Poisson distribution was used to determine the 95% CI for these calculated rates.

Generalized Estimating Equations (GEE) negative binomial regression model was built to identify the baseline and poststent factors associated with the number of hospital admissions [33]. The logarithm of the duration of follow-up in person-years of each subject was modeled as an offset and subjects were clustered by the admission institution. The outcome variable was the number of hospital admissions. Patient age, sex, and comorbidity were retained in the model and backward stepwise reduction was used to identify factors independently associated with the number of hospital admissions.

All statistical tests were two-sided and *p* values less than 0.05 were considered significant. All analyses were performed using SAS 9.1 for Unix (SAS Institute, Cary, USA) and R for Unix [34].

*Ethics*. The Research Ethics Board at Sunnybrook Health Sciences Centre and the Office of Research Ethics at the University of Toronto approved the study.

## 4. Results

### 4.1. Study Cohort, Physician Specialty, and Institutions

From April 1, 2000, to March 30, 2009, 374 individuals were identified who had both a stent inserted (endoscopic 145 and radiologic 229) and a previous or concurrent diagnosis of CRC. Of those, 149 individuals were excluded in the radiologic stent insertion group due to stent insertion outside of colon/rectum (61) and missing DAD CCP and CCI procedural codes (88), leaving 225 patients for the final analysis.

The baseline characteristics of the cohort are summarized in Table 1. In brief, 2/3 of the cohort consisted of males with a median age of 69. Over 2/3 of the patients had metastatic disease at the time of stent insertion but were otherwise relatively healthy. Patient level factors did not differ significantly by mode of stent insertion.

**Figure 1:**
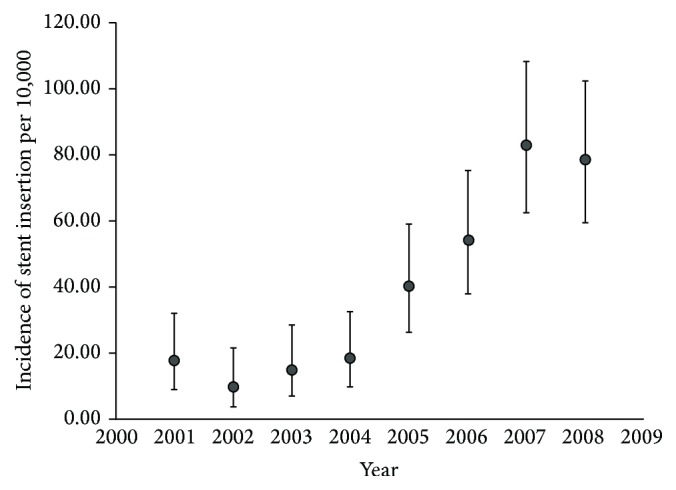
Annual age and sex adjusted incidence of stent insertion per 10,000 patients with colorectal cancer. Patients receiving stents in 2000 and 2009 were not included as data was not available for the entire calendar year.

Overall, the incidence of stent insertion among individuals with CRC increased over the study period (Figure 1). The majority of stents were inserted by gastroenterologists followed by radiologists and then surgeons (Table 2). Overall 54.4% of physicians inserted two or more stents over the study period and gastroenterologists inserted more stents per physician (Table 2) than did other specialties.

A similar number of stents were inserted in both the academic (49%) and community (51%) institutions. In the community hospitals, stents were significantly more likely to be inserted endoscopically rather than radiologically (Table 1).

### 4.2. Health Outcomes

**Figure 2:**
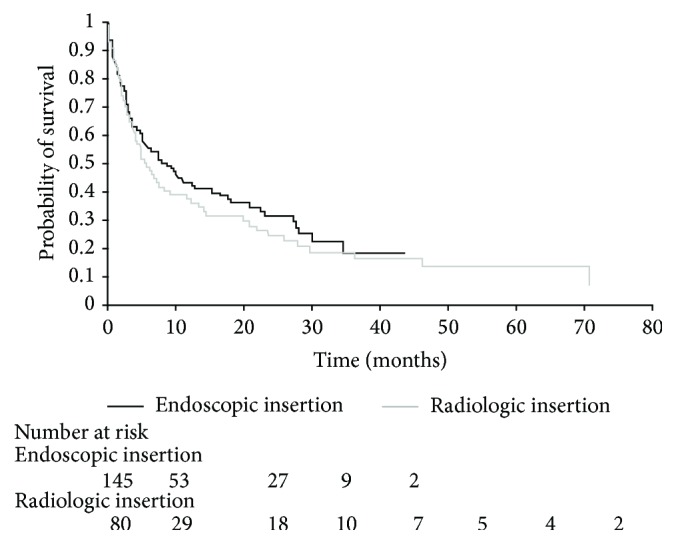
Kaplan-Meier survival curve for overall survival in patients with colorectal cancer managed with colorectal stents stratified by modality of stent insertion.

By the end of the follow-up period, 153 (68%) of patients had died with a median survival of 199 days (interquartile range, IQR, 69–834 days). Survival was worse for patients with metastatic disease (HR 2.3, 95% CI 1.6–3.5) compared to those without; however mode of stent insertion did not significantly affect survival (HR 0.85, 95% CI 0.62–1.2) (Figure 2).

By the end of the follow-up period, 85 (37%) patients had a total of 89 additional procedures after the index stent insertion. Twenty-five restenting and/or dilatation procedures and 64 surgical procedures were performed. Of the former, 17 patients required repeat stenting alone, 6 patients required dilatation alone, and two patients required both stenting and dilatation.

Of the 64 operations, 26 were resections with primary anastomosis, 23 were resections with stoma creation, and 14 were stoma creations alone (Table 3). Of the 66 patients without metastatic disease, 23 (35%) went on to have surgery compared to 41 of 159 (26%) of patients with metastatic disease. Patients without metastatic disease had a tendency to have potentially curative surgery (resection with or without stoma) while patients with metastatic disease were more likely to have potentially palliative surgery (stoma creation without resection) (Table 3).

**Figure 3:**
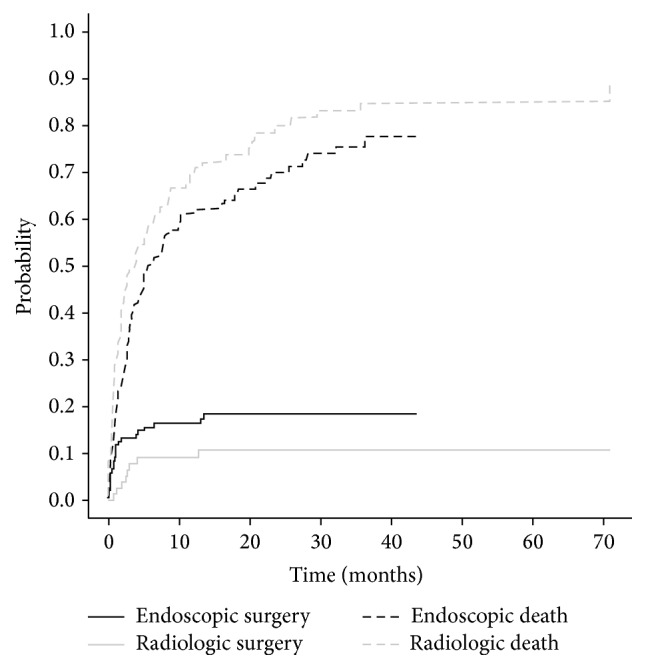
Cumulative incidence curve for surgery in patients with colorectal cancer managed with colorectal stents accounting for competing risk of death, stratified by mode of stents insertion.

Figure 3 models the time to nondeath events, stratified by mode of stent insertion. In this cumulative incidence plot, the height of the lower curves is the cumulative incidence for surgery at year *t* (Figure 3). The distance between the top curves and the lower curves at *t* years is the surgery-free mortality cumulative incidence at this time. One minus the top curves is the surgery-free survival probability at year *t*. From the results of the cause-specific hazard regression model for surgery, the rate of surgery is lower among patients with metastatic disease (HR 0.14, 95% CI 0.06–0.32, *p* < 0.0001) and patients who received a radiologic stent (HR 0.43, 95% CI 0.20–0.92, *p* < 0.03) (Table 4). The results from the plots of the cause-specific hazard regression models for the other nondeath outcomes are not reported as they either did not converge (time to repeat stent or dilation) or added no new information (time to stoma).

### 4.3. Health Care Encounters

During the study follow-up period, 489 emergency department visits were recorded for the study cohort yielding an emergency department visit rate of 2.4 per person-year (95% CI 2.2–2.7). Similarly, there were 456 hospital admissions over the study period (2.3 hospital admissions per person-year (95% CI 2.1–2.5)), accounting for 19 inpatient days per person-year (95% CI 18-19). Most admissions were cancer related, procedure related, or for palliation. Fifty-four patients required a hospital admission for obstruction and/or perforation (48 obstructions alone, 4 perforations alone, and 2 obstructions with perforation); 37 had further intervention while 17 had no further active treatment. In the GEE binomial regression model, three factors were identified to be independently associated with hospital admission: (1) presence of metastatic disease at the time of stent insertion, (2) lack of chemotherapy treatment after stent insertion, and (3) stoma surgery after stent insertion.

## 5. Discussion

In this first population-based study of CR stenting for CRC obstruction, we found that patients who received stents were predominantly older, otherwise healthy men who had metastatic disease. Volume of stent insertions increased over the study period but overall stent use for CRC-related obstruction was low. More stents were placed endoscopically than radiologically but the mode of stent insertion did not seem to be associated with patient level factors. Stents appeared more likely to be used as a means of palliation rather than as a bridge to surgery. Median survival after stent insertion was slightly over 6 months and during this time the rates of major medical events (reintervention or admission to hospital) were low.

The increase in the use of CR stents for CRC in Ontario between 2000 and 2008 is most likely a reflection of growing physician and hospital familiarity and experience with this technology. However, the overall use of stents for CRC obstruction appears to be very low. In 2008, an estimated 8000 individuals were diagnosed with CRC in the province of Ontario [1]; of these 15% (approximately 1200) are estimated to have presented with bowel obstruction [3, 5, 6]. We found that only 55 stents were inserted in patients with CRC in that year, suggesting that only approximately 5% of patients with obstruction due to CRC were managed with a stent. This study was not designed to determine the reasons for this low utilization, but we hypothesize that lack of physician expertise and the upfront cost associated with stent use could both contribute. Further study is required to identify reasons for the low utilization and to determine if the observed rate is appropriate, particularly as it has been shown that CR stents are cost effective [35, 36].

Although more stents were placed endoscopically than radiologically, no significant differences were noted in patient or disease factors. However, we found that endoscopic deployment was more frequently used in community hospitals, while radiologic stents were more likely to be placed in academic institutions, perhaps indicating that institutional factors are an important driver of the mode of stent insertion.

Stents seemed to be used more often as a means of palliation rather than a bridge to curative surgery as most patients had metastatic disease at the time of stent insertion. The fairly short poststent median survival of 199 days supports this conclusion, as it is in keeping with previously published median survival times of 119 to 150 days in advanced stage CRC palliated with colonic stents [37, 38]. While results from recent prospective trials of stents in acute malignant obstruction have been mixed, similar literature on the role of stenting in palliative patients is limited [21]. Poststent surgery was less likely in those with metastatic disease compared to those without when adjusted for the competing risk of death. Further research is needed to clarify the benefit of stents in palliative patients given the higher utilization we have observed in this population and the recent disappointing literature on stents used for acute obstruction due to CRC [22–24].

Just over one-third of patients required a second intervention after the index stent insertion with low overall rates of surgery (28%) and stoma creation (16%). These findings are comparable to a large cohort of 223 palliative and bridge to surgery patients from the Mayo Clinic where 65% and 85% of patients, respectively, did not require additional intervention or surgery [20]. Patients who had a stent placed radiologically were less likely to go onto surgery, after adjustment for the competing risk of death and presence of metastatic disease. This finding could reflect better performance of radiologically placed stents in palliative patients or could indicate a preference for endoscopically placed stents when the intention is to bridge to surgery. The rate of emergency department visits and hospital admissions per person-year appears low, as was the average number of days in hospital per person-year, indicating that patients were able to spend the majority of their remaining time alive out of hospital.

This study was intended to be primarily descriptive and hypothesis generating. As this was a population-based study, we were able to describe the utilization of colonic stenting for CRC in usual practice. A major advantage of this approach over previously published literature is that it minimizes the effects of a single center bias. However, there are limitations to using administrative data in Ontario, including the lack of information about CRC stage and about the clinical decision making process. For these two reasons, we felt that a comparison of our population to a control group that did not have a stent placed was inappropriate, as we could not adequately adjust for confounders.

In summary, this is the first study to describe colonic stenting in patients with CRC in a real-world setting. This approach yields observations that would not arise from single center prospective or controlled studies, such as the apparent low rate of CR stenting in this population, which should be explored further here and compared to other jurisdictions. In addition, we have identified key variables (physician specialty, physician volume, mode of stent insertion, and hospital volume) that may affect outcomes and patient-centered outcomes (e.g., intervention rate and hospital admissions) that also merit further investigation. Finally, economic analyses of costs associated with use of stents in usual practice would be informative and would provide guidance for practitioners, hospital administrators, and health care decision makers.

## Figures and Tables

**Table 1:** Patient and institution characteristics overall and by mode of index stent insertion for persons with colorectal cancer who received colorectal stents between April 1, 2000, and March 31, 2009.

Variable	Total	Endoscopy	Radiology	*p* value^*∗*^
	(*N* = 225)	(*N* = 145)	(*N* = 80)	
	*n* (%)	*n* (%)	*n* (%)	
Median age at stent insertion in years (IQR)	69 (59–77)	71 (59–78)	68 (57–75)	0.17
Sex				
Female	79 (35%)	47 (32%)	32 (40%)	0.25
Male	146 (65%)	98 (68%)	48 (60%)	
Comorbidity score^*∗∗*^				
0	147 (66%)	99 (68%)	48 (60%)	0.43
1	31 (14%)	16 (11%)	15 (19%)	
2	20 (8.9%)	13 (9.0%)	7 (8.8%)	
3	26 (11%)	17 (12%)	9 (11%)	
Income quintile				
1 (lowest)	32 (14%)	21 (14%)	11 (14%)	0.54
2	49 (22%)	27 (19%)	22 (27%)	
3	44 (20%)	30 (21%)	14 (18%)	
4	42 (19%)	26 (18%)	16 (20%)	
5 (highest)	57 (25%)	40 (28%)	17 (21%)	
Metastases				
Yes	159 (71%)	103 (71%)	56 (70%)	0.87
No	66 (29%)	42 (29%)	24 (30%)	
Median time from CRC diagnosis to index stent insertion in days (IQR)	32 (6–347)	28 (7–343)	44 (5–369)	0.44
Chemotherapy prior to index stent insertion				
Yes	53 (24%)	32 (22%)	21 (26%)	0.50
No	172 (76%)	113 (78%)	59 (74%)	
Type of hospital				
Academic	110 (49%)	63 (43%)	47 (59%)	0.03
Community	115 (51%)	82 (57%)	33 (41%)	

^*∗*^ *p* value for comparison between modalities.

^*∗∗*^Derived using a modified version of the Charlson/Deyo Comorbidity Index score which excluded cancer diagnosis and presence of metastases.

**Table 2:** Number of physicians and colonic stents by physician specialty in unique patients with colorectal cancer receiving their index stent between April 1, 2000, and March 31, 2009.

Physician specialty	Number of unique physicians	Total number of index colonic stents placed by specialty	Median number of index colonic stents per physician specialty (IQR)
Gastroenterology	27	130	3 (1–7)
Radiology	31	78	1 (1–3)
General surgery	9	17	1 (1-2)

**Table 3:** Type of surgery after index colorectal stent insertion in patients with colorectal cancer by baseline metastatic status from April 1, 2000, to March 31, 2009.

Type of surgery	Patients without metastases	Patients with metastases	*p* value
	(*n* = 66)	(*n* = 159)	
	*n* (%)	*n* (%)	
Resection with primary anastomosis	12 (52)	14 (34)	0.16
Resection with stoma creation	9 (39)	14 (34)	0.69
Stoma creation only without resection	2 (9)	12 (29)	0.06
Unknown	0 (0)	1 (2)	N/A

N/A: not assessed.

**Table 4:** Relationship between time to surgery and patient age, sex, metastases at presentation, and types of stent based on a multivariate regression model using a cause-specific hazard approach to accommodate for the competing risk of death.

Variable	Hazard ratio (95% CI)	*p* value
Age	0.97 (0.95–1.00)	0.03
Sex		
Female	Reference	0.18
Male	0.78 (0.38–1.59)	
Metastases		
No	Reference	0.001
Yes	0.14 (0.06–0.32)	
Stent type		
Endoscopic	Reference	0.03
Radiologic	0.43 (0.20–0.92)	

## Additional Points

(i) Colorectal stents seem to be infrequently used in the management of CRC obstruction; the reasons for this observation are not known. (ii) When used, in usual practice colorectal stents appear to be used more often for the palliative management of CRC obstruction. (iii) In usual practice, patients with CRC obstruction who are managed with colorectal stents appear to have good health outcomes and low rates of subsequent health care utilization.

## References

[1] Canadian Cancer Society http://publications.gc.ca/collections/collection_2011/statcan/CS2-37-2011-eng.pdf.

[2] National Cancer Institute http://seer.cancer.gov/.

[3] Phillips R. K. S., Hittinger R., Fry J. S., Fielding L. P. (1985). Malignant large bowel obstruction. *British Journal of Surgery*.

[4] Mella J., Biffin A., Radcliffe A. G., Stamatakis J. D., Steele R. J. C. (1997). Population-based audit of colorectal cancer management in two UK health regions. *British Journal of Surgery*.

[5] Serpell J. W., McDermott F. T., Katrivessis H., Hughes E. S. R. (1989). Obstructing carcinomas of the colon. *British Journal of Surgery*.

[6] Minopoulos G. I., Lyratzopoulos N., Efremidou H. I., Romanidis K., Koujoumtzi I., Manolas K. J. (2004). Emergency operations for carcinoma of the colon. *Techniques in Coloproctology*.

[7] Irvin T. T., Greaney M. G. (1977). The treatment of colonic cancer presenting with intestinal obstruction. *British Journal of Surgery*.

[8] Runkel N. S., Schlag P., Schwarz V., Herfarth C. (1991). Outcome after emergency surgery for cancer of the large intestine. *British Journal of Surgery*.

[9] Tekkis P. P., Kinsman R., Thompson M. R., Stamatakis J. D. (2004). The association of coloproctology of Great Britain and Ireland study of large bowel obstruction caused by colorectal cancer. *Annals of Surgery*.

[10] Koruth N. M., Hunter D. C., Krukowski Z. H., Matheson N. A. (1985). Immediate resection in emergency large bowel surgery: a 7 year audit. *British Journal of Surgery*.

[11] Spinelli P., Dal Fante M., Mancini A. (1992). Self-expanding mesh stent for endoscopic palliation of rectal obstructing tumors: a preliminary report. *Surgical Endoscopy*.

[12] Dohmoto M., Hünerbein M., Schlag P. M. (1997). Application of rectal stents for palliation of obstructing rectosigmoid cancer. *Surgical Endoscopy*.

[13] Tejero E., Mainar A., Fernandez L., Tobío R., De Gregorio M. A. (1994). New procedure for the treatment of colorectal neoplastic obstructions. *Diseases of the Colon & Rectum*.

[14] Karoui M., Charachon A., Delbaldo C. (2007). Stents for palliation of obstructive metastatic colon cancer: impact on management and chemotherapy administration. *Archives of Surgery*.

[15] Dasari B. V. M., Tan C. J., Gardiner K. (2012). Systematic review and meta-analysis of randomized clinical trials of self-expanding metallic stents as a bridge to surgery versus emergency surgery for malignant left-sided large bowel obstruction. *British Journal of Surgery*.

[16] Mainar A., De Gregrio Ariza M. A., Tejero E. (1999). Acute colorectal obstruction: treatment with self-expandable metallic stents before scheduled surgery—results of a multicenter study. *Radiology*.

[17] Camúñez F., Echenagusía A., Simó G., Turégano F., Vázquez J., Barreiro-Meiro I. (2000). Malignant colorectal obstruction treated by means of self-expanding metallic stents: effectiveness before surgery and in palliation. *Radiology*.

[18] Ho K.-S., Quah H.-M., Lim J.-F., Tang C.-L., Eu K.-W. (2012). Endoscopic stenting and elective surgery versus emergency surgery for left-sided malignant colonic obstruction: a prospective randomized trial. *International Journal of Colorectal Disease*.

[19] Sebastian S., Johnston S., Geoghegan T., Torreggiani W., Buckley M. (2004). Pooled analysis of the efficacy and safety of self-expanding metal stenting in malignant colorectal obstruction. *The American Journal of Gastroenterology*.

[20] Small A. J., Coelho-Prabhu N., Baron T. H. (2010). Endoscopic placement of self-expandable metal stents for malignant colonic obstruction: long-term outcomes and complication factors. *Gastrointestinal Endoscopy*.

[21] Sagar J. (2011). Colorectal stents for the management of malignant colonic obstructions. *Cochrane Database of Systematic Reviews*.

[22] Pirlet I. A., Slim K., Kwiatkowski F., Michot F., Millat B. L. (2011). Emergency preoperative stenting versus surgery for acute left-sided malignant colonic obstruction: a multicenter randomized controlled trial. *Surgical Endoscopy and Other Interventional Techniques*.

[23] van Hooft J. E., Bemelman W. A., Oldenburg B. (2011). Colonic stenting versus emergency surgery for acute left-sided malignant colonic obstruction: a multicentre randomised trial. *The Lancet Oncology*.

[24] van Hooft J. E., Fockens P., Marinelli A. W. (2008). Early closure of a multicenter randomized clinical trial of endoscopic stenting versus surgery for stage IV left-sided colorectal cancer. *Endoscopy*.

[25] Fiori E., Lamazza A., De Cesare A. (2004). Palliative management of malignant rectosigmoidal obstruction. Colostomy vs. endoscopic stenting. A randomized prospective trial. *Anticancer Research*.

[26] Robles S. C., Marrett L. D., Aileen Clarke E., Risch H. A. (1988). An application of capture-recapture methods to the estimation of completeness of cancer registration. *Journal of Clinical Epidemiology*.

[27] Charlson M. E., Pompei P., Ales K. L., MacKenzie C. R. (1987). A new method of classifying prognostic comorbidity in longitudinal studies: development and validation. *Journal of Chronic Diseases*.

[28] Deyo R. A., Cherkin D. C., Ciol M. A. (1992). Adapting a clinical comorbidity index for use with ICD-9-CM administrative databases. *Journal of Clinical Epidemiology*.

[29] Ouellette J. R., Small D. G., Termuhlen P. M. (2004). Evaluation of Charlson-Age Comorbidity Index as predictor of morbidity and mortality in patients with colorectal carcinoma. *Journal of Gastrointestinal Surgery*.

[30] Nenshi R., Baxter N., Kennedy E., Urbach D. R., Simunovic M., Schultz S. E. (2008). Surgery for colorectal cancer. *Cancer Surgery in Ontario: ICES Atlas*.

[31] Rabeneck L., Paszat L. F., Rothwell D. M., He J. (2005). Temporal trends in new diagnoses of colorectal cancer with obstruction, perforation, or emergency admission in Ontario: 1993–2001. *The American Journal of Gastroenterology*.

[32] Putter H., Fiocco M., Gekus R. B. (2007). Tutorial in biostatistics: competing risks and multi-state models. *Statistics in Medicine*.

[33] McCullagh P., Nelder J. L. (1989). *Generalized Lineal Models*.

[34] R Development Core Team (2009). *A Language and Environment for Statistical Computing*.

[35] Singh H., Latosinsky S., Spiegel B. M. R., Targownik L. E. (2006). The cost-effectiveness of colonic stenting as a bridge to curative surgery in patients with acute left-sided malignant colonic obstruction: a Canadian perspective. *Canadian Journal of Gastroenterology*.

[36] Siddiqui A., Khandelwal N., Anthony T., Huerta S. (2007). Colonic stent versus surgery for the management of acute malignant colonic obstruction: a decision analysis. *Alimentary Pharmacology and Therapeutics*.

[37] Law W. L., Choi H. K., Chu K. W. (2003). Comparison of stenting with emergency surgery as palliative treatment for obstructing primary left-sided colorectal cancer. *British Journal of Surgery*.

[38] Xinopoulos D., Dimitroulopoulos D., Theodosopoulos T. (2004). Stenting or stoma creation for patients with inoperable malignant colonic obstructions? Results of a study and cost-effectiveness analysis. *Surgical Endoscopy and Other Interventional Techniques*.

